# Top‐down Surfactant‐Free Synthesis of Supported Palladium‐Nanostructured Catalysts

**DOI:** 10.1002/smsc.202300241

**Published:** 2024-01-11

**Authors:** Christian M. Schott, Peter M. Schneider, Kais Sadraoui, Kun‐Ting Song, Batyr Garlyyev, Sebastian A. Watzele, Jan Michalička, Jan M. Macak, Arnaud Viola, Frédéric Maillard, Anatoliy Senyshyn, Johannes A. Fischer, Aliaksandr S. Bandarenka, Elena L. Gubanova

**Affiliations:** ^1^ Physics of Energy Conversion and Storage Technical University of Munich James Franck Str. 1 Garching 85748 Germany; ^2^ Central European Institute of Technology Brno University of Technology Purkynova 123 Brno 61200 Czech Republic; ^3^ Center of Materials and Nanotechnologies University of Pardubice Nam. Cs. Legii 565 Pardubice 53002 Czech Republic; ^4^ Université Grenoble Alpes Université Savoie Mont Blanc CNRS Grenoble INP LEPMI Grenoble 38000 France; ^5^ Heinz Maier‐Leibnitz‐Zentrum (MLZ) TUM Lichtenbergstr. 1 Garching 85748 Germany; ^6^ Catalysis Research Center TUM Ernst‐Otto‐Fischer‐Str. 1 Garching 85748 Germany

**Keywords:** electrochemical erosion, hydrogen embrittlement, hydrogen evolution reaction, nanoparticles, palladium

## Abstract

Nanostructured palladium (Pd) is a universal catalyst that is widely used in applications ranging from catalytic converters of combustion engine cars to hydrogenation catalysts in industrial processes. Standard protocols for synthesizing such nanoparticles (NPs) typically use bottom‐up approaches. They utilize special and often expensive physical techniques or wet‐chemical methods requiring organic surfactants. These surfactants should often be removed before catalytic applications. In this article, the synthesis of Pd NPs immobilized on carbon support by electrochemical erosion without using any surfactants or toxic materials is reported. The Pd NPs synthesis essentially relies on a Pd bulk pretreatment, which causes material embrittlement and allows the erosion process to evolve more efficiently, producing homogeneously distributed NPs on the support. Moreover, the synthesized catalyst is tested for hydrogen evolution reaction. The activity evaluations identify optimal synthesis parameters related to the erosion procedure. The electrocatalytic properties of the Pd NPs produced with sizes down to 6.4 ± 2.9 nm are compared with a commercially available Pd/C catalyst. The synthesized catalyst outperforms the commercial catalyst within all properties, like specific surface area, geometric activity, mass activity, specific activity, and durability.

## Graphical abstract

A surfactant‐free top‐down approach, called “electrochemical erosion”, allows the fabrication of palladium (Pd) nanoparticles (NPs) supported on Vulcan carbon. Crucially, a Pd wire pretreatment is identified as the essential step to synthesize NPs with sizes below 10 nm. The synthesized Pd/C catalysts are thoroughly analyzed for their structure, morphology, chemical composition, and electrochemical activity toward the hydrogen evolution reactions.

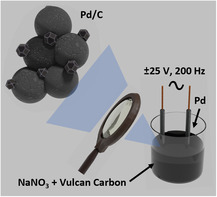

## 1 Introduction

Palladium (Pd) serves as a universal catalyst for electrochemical and non‐electrochemical reactions. For instance, it catalyzes the functionalization of propene^[^ ^1^, ^2^ ^]^ with high industrial relevance and the direct oxidation of methane.^[^ ^3^ ^]^ Alternatively, Pd electrochemically generates hydrogen peroxide,^[^ ^4^ ^]^ accessing a highly relevant oxidizing agent in organic synthesis. Aside from fuel generation, Pd and Pd‐based alloys compete with state‐of‐the‐art catalysts in direct liquid‐fed fuel cells and proton‐exchange membrane fuel cells for fuel consumption reactions.^[^ ^5^, ^6^ ^]^ The former fuel cell system relies on the oxidation reaction of small organic molecules like methanol,^[^ ^7^, ^8^ ^]^ ethanol,^[^ ^9^ ^]^ and formic acid.^[^ ^10^ ^]^ The latter involves the hydrogen oxidation reaction (HOR) and oxygen reduction reaction (ORR).^[^ ^11^, ^12^, ^13^ ^]^ Independent of the catalyzed reaction, the efficiency of the Pd catalyst strongly depends on the shape and size of the nanoparticles (NPs). Synthesis methods fabricate and homogeneously disperse NPs on a suitable support material via physical, biological, or chemical mechanisms.^[^ ^14^ ^]^ Among them, surfactant‐free methods attract attention since these organic additives contaminate the metallic NP surface and thus decrease the catalytic performance.^[^ ^15^, ^16^ ^]^ To avoid this loss of activity, we present a surfactant‐free, cheap, and upscalable top‐down (TD) electrochemical method, which we call electrochemical erosion, in the scope of our work. The technique applies an alternating potential to metal wires, generating NPs at the metal surface of the wires immersed in an electrolyte suspension. The groups of Hersbach and Koper showed that the electrolyte, especially the involved cation, plays a crucial role in NP formations.^[^ ^17^ ^]^ Especially alkali‐metal (AM)‐comprised electrolytes (AMOH, where AM = Li, Na, K, and Cs) showed promising results, but NPs formation also occurred for Ca(NO_3_)_2_, Na_2_SO_4_, CaCl_2_, and NH_4_^+^ cations containing electrolytes.^[^ ^18^ ^]^ Next to the electrolyte, the synthesis suspension consists of a dispersed support material, corresponding mostly to Vulcan carbon due to its high surface area, excellent electronic conductivity, and/or sufficient chemical stability.^[^ ^19^, ^20^ ^]^ Several groups in the field of electrocatalysis elucidated electrochemical erosion to fabricate nanostructured catalysts based on different metals. Among others, those include Rh, Bi, Sn, Pb, Au, and Cu, synthesized by the groups of Li^[^ ^21^, ^22^, ^23^, ^24^ ^]^ and Koper.^[^ ^18^, ^25^, ^26^, ^27^, ^28^ ^]^ Our group primarily focused on Pt alloys, like Pt_x_Pr^[^ ^29^ ^]^ and pure Pt.^[^ ^30^ ^]^ These Pt NPs exhibit better ORR activity than commercial catalysts.^[^ ^31^ ^]^

The electrochemical erosion approach allows to produce Pt‐containing NPs with a single‐digit nanometric diameter. At such particle dimensions, size and shape modifications can essentially dictate their adsorption properties, influencing the activity toward specific reactions. Driven by elucidating this size and shape effect of Pt NPs, Garlyyev et al. explored shape and size variations via electrochemical erosion.^[^ ^30^ ^]^ Nevertheless, in the past, Pd seemed inapplicable for these types of techniques. Yanson et al.^[^ ^25^ ^]^ attributed this failure to the formation of a hydride layer at the Pd surface during electrochemical erosion. Later, Feng et al.^[^ ^18^ ^]^ reported the synthesis of Pd nanostructures in 0.3 м CaCl_2_, but only with the indispensable presence of the surfactant polyvinylpyrrolidone (PVP). However, organic additives are known to contaminate the surface of the catalyst, which leads to a decrease in the catalytic performance of the synthesized catalyst. Furthermore, it is known that synthesizing nanostructures by electrochemical erosion without using surfactants leads to large agglomerations and highly polydisperse particles.^[^ ^18^, ^25^ ^]^ Despite these challenges, we present in this work the first surfactant‐free synthesis of Pd NPs by electrochemical erosion, which leads to the production of single‐digit nanometer‐sized Pd NPs, homogenously distributed on Vulcan carbon, that significantly outperform a commercial reference Pd/C catalyst toward the hydrogen evolution reaction (HER).

## 2 Results and Discussion

### 2.1 Synthesis of Pd NPs

**Figure 1:**
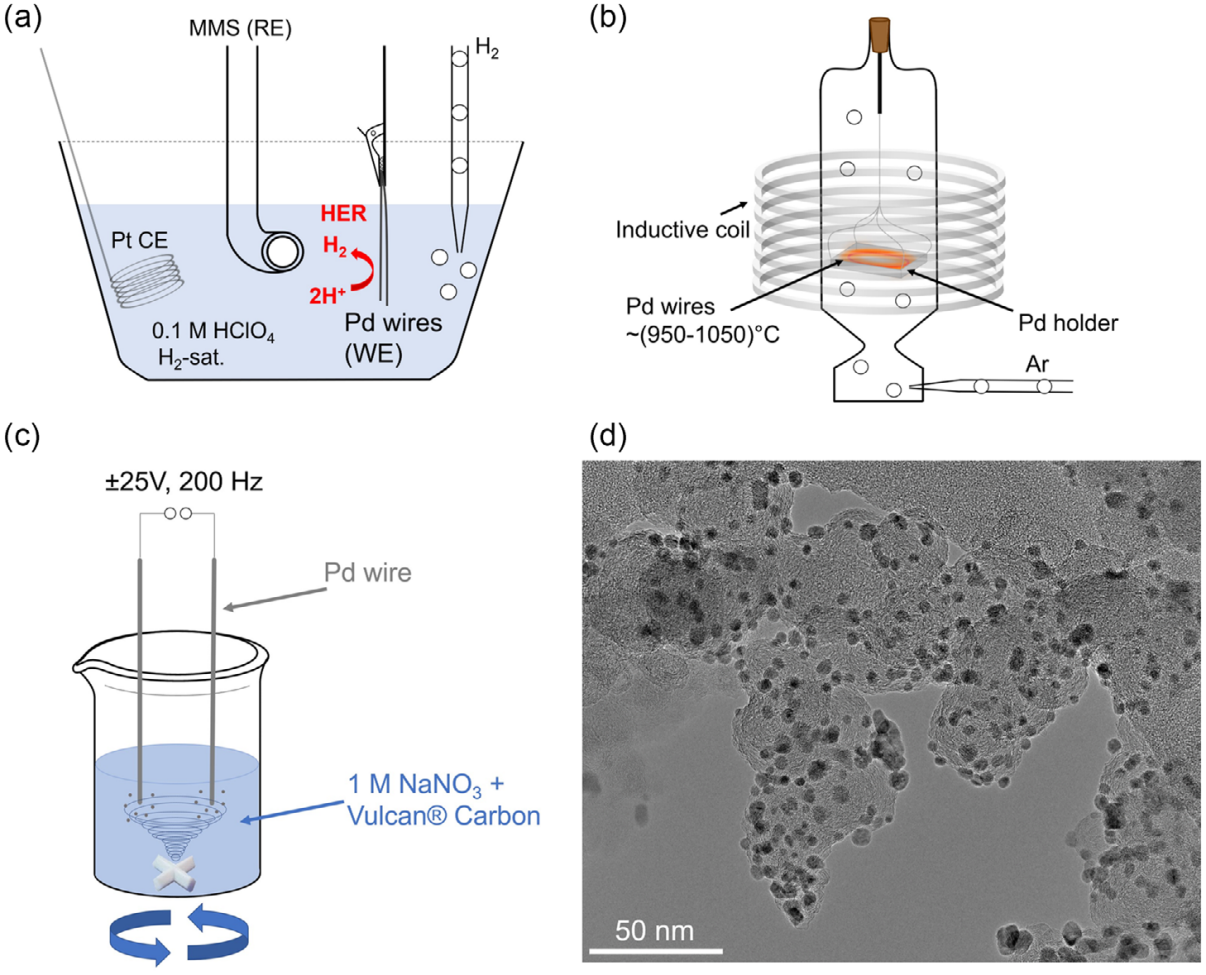
a) Schematic of the hydrogen evolution reaction (HER) pretreatment of Pd wires immersed in a three‐electrode cell containing H_2_‐saturated 0.1 м HClO_4_ electrolyte. The Pd wires serve as a working electrode (WE) to produce molecular H_2_ during cyclic voltammetry. b) Schematic of the subsequent annealing pretreatment, displaying the annealed Pd at ≈950–1050 °C on a Pd holder in an inductive heater under an Ar atmosphere. c) Schematic of the top‐down (TD) electrochemical erosion approach. Nanoparticles (NPs) formed due to the application of a ± 25 V, 200 Hz sinusoidal potential signal in a suspension containing 1 м NaNO_3_ and dispersed Vulcan carbon. d) A transmission electron microscope (TEM) image of the Pd/C catalyst after synthesis.

To fabricate Pd NPs with a diameter below 10 nm, we developed a surfactant‐free synthesis procedure. Next to electrochemical erosion, the method involves two crucial steps, as depicted in **Figure** **1**a,b, which showcase the optimized two‐step pretreatment approach of Pd wires employed in this study. The first step, displayed in Figure 1a, alters the structural properties of Pd due to the formation of palladium hydride (PdH_x_), e.g., during HER in acidic media. H atoms diffuse into the crystal structure of Pd and occupy octahedral voids,^[^ ^32^ ^]^ which stresses the crystal structure and subsequently deform and embrittle the Pd wire.^[^ ^33^, ^34^, ^35^, ^36^ ^]^ The embrittlement is not restricted to the wire surface since it is well known that hydrogen diffuses into subsurface and bulk regions.^[^ ^37^ ^]^ The second step, illustrated in Figure 1b, modifies the structural properties even further via an annealing and cooling treatment in an Ar atmosphere at ≈950–1050 °C. First, the annealing secures the absorbed hydrogen leaving the Pd bulk. Second, heat treatment with succeeding cooling of Pd forms terrace arrangements and deep grooves or cracks at grain boundaries located on the surface of the Pd wires.^[^ ^38^ ^]^ Similarly, Yule et al.^[^ ^39^ ^]^ performed flame annealing with subsequent quenching in deionized water. They identified the resulting distortions in the grain boundaries of the Pd structure as hot spots for hydrogen absorption through scanning electrochemical cell microscopy techniques. Due to those hot spots, Pd wires absorb hydrogen more dominantly during HER, resulting in a bigger embrittlement effect originating from PdH_x_ formation. Accordingly, to guarantee a maximal state of embrittlement, we individually repeated the HER and annealing pretreatment up to three times. Scanning electron microscopy (SEM) experiments clarified the effect of the two‐step pretreatments by comparing the morphology of the Pd wire before and after the process, as well as after the erosion procedure, as illustrated in Figure S1, Supporting Information. After performing the two‐step pretreatment, we electrochemically eroded the rough Pd wires by immersing them in a suspension and applying an alternating potential signal. Figure 1c displays a scheme of the electrochemical erosion technique, which involves multiple parameters that are likely to affect the synthesized Pd NPs. These parameters include the type of wire pretreatment, NaNO_3_ concentration in the electrolyte, and the applied erosion signals, such as frequency and amplitude. Initially, we conducted the two‐step pretreatment once and varied the frequency of a ± 25 V applied sinusoidal voltage signal from 20 to 200 Hz. A detailed summary of the obtained results can be found in Supporting Information. Nevertheless, Pd NPs synthesized via a three‐time‐repeated pretreatment with a ± 25 V, 200 Hz applied sinusoidal voltage signal in 1 м NaNO_3_ electrolyte exhibited more promising results. The transmission electron microscopy (TEM) image in Figure 1d depicts a rough overview of the synthesized Pd NPs and their homogeneous distribution on the Vulcan carbon support. In the following part, we more profoundly elucidate their structural and compositional properties. The conducted characterizations include TEM, which more accurately describes NP dispersion on the support material. By using high‐resolution TEM (HRTEM), scanning TEM (STEM) and STEM with energy‐dispersive X‐ray spectroscopy (STEM–EDX), we investigated their size, shape, and chemical composition, respectively.

### 2.2 Catalyst Characterization by TEM, STEM, HRTEM, STEM–EDX, and X‐ray Photoelectron Spectroscopy

**Figure 2:**
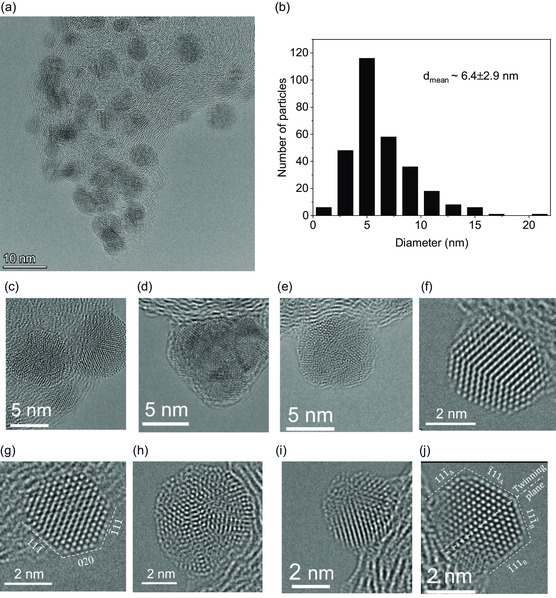
TEM investigation of the Pd/C catalyst synthesized in 1 м NaNO_3_ with different resolutions. a) A TEM overview image. b) Histogram of the diameter distribution and mean diameter of the synthesized Pd nanoparticles (NPs) on carbon support. c–j) High‐resolution TEM (HRTEM) images of single Pd particles with atomic resolution. For (g and j), the Miller indexes of the respective lattice planes were added since the NPs are well oriented to the (011) zonal axis.

**Figure 3:**
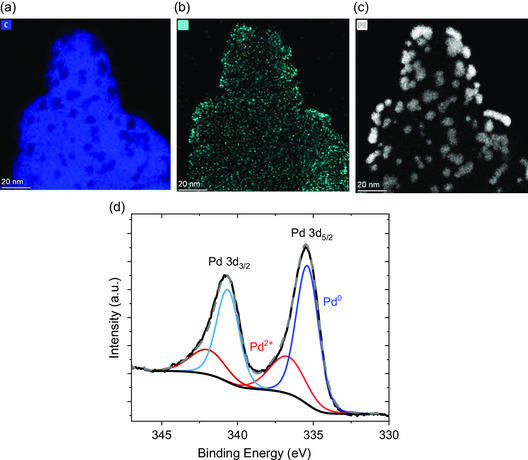
Scanning transmission electron microscopy with energy‐dispersive X‐ray spectroscopy (STEM–EDX) of the Pd/C catalyst synthesized in 1 м NaNO_3_ with elemental mapping of a) C–K, b) O–K, and c) Pd–L. d) X‐ray photoelectron spectroscopy (XPS) spectrum of the Pd/C catalyst. The Pd 3d peaks indicate the presence of Pd^0^ and Pd^2+^, corresponding to metallic and oxidized Pd states, respectively.

**Figure** **2**a provides a magnified, atomically resolved view of the overview in Figure 1d. The high resolution validates the homogeneous distribution of the Pd NPs, even though certain areas exhibit a slightly higher density of NPs. Figure 2b presents a Pd NP size distribution and a mean average diameter of 6.4 ± 2.9 nm, as measured from TEM and STEM images. In addition, Figure 2c–j displays HRTEM images of single Pd NPs. The particles reveal crystalline structure, and their sharp edges suggest a nonspherical but rather polyhedrical shape. We emphasize that it is difficult to make conclusive results of the observed NPs polyhedron types from the images; however, they appear to form cubical up to complex polyhedron shapes (in the case of larger particles), as indicated by the different number of facets revealed in the recorded images. Next to the TEM and HRTEM imaging of the structure, we used EDX in STEM to create elemental maps of the Pd/C catalyst. The investigated elements include carbon, oxygen, and palladium, as shown in **Figure** **3**a–c. Figure 3b reveals a homogeneous distribution of oxygen in the EDX map, which may arise either from the H_2_O_2_ pretreatment of the Vulcan XC72R or from atmospheric oxygen, which oxidized its surface and formed oxygen‐containing functional groups at the surface.^[^ ^40^ ^]^ The oxygen signals do not correlate with the positions of the Pd NPs, depicted in Figure 3c; only at the carbon edges some of the positions coincide. We highlight that detecting oxygen by EDX proves problematic if the oxygen is present in small concentrations, such as a thin oxidized shell around NPs. Therefore, X‐ray photoelectron spectroscopy (XPS) measurements were conducted to identify the oxide contribution of the Pd/C catalyst more accurately. Figure 3d illustrates the analysis of 3d_3/2_ and 3d_5/2_ doublet of Pd^0^ and Pd^2+^ spectra, which refers to metallic and oxidized Pd, respectively. Calculating the peak areas, we estimated the atomic content of metallic Pd^0^ at 70% and Pd^2+^ at 30%. It is highly probable that a significant amount of the Pd^2+^ forms during the erosion process due to strong anodic polarization, which significantly promotes oxygen evolution.

### 2.3 Study of the Electrolyte Concentration on the Pd NPs Synthesis Using X‐ray Diffraction

**Figure 4:**
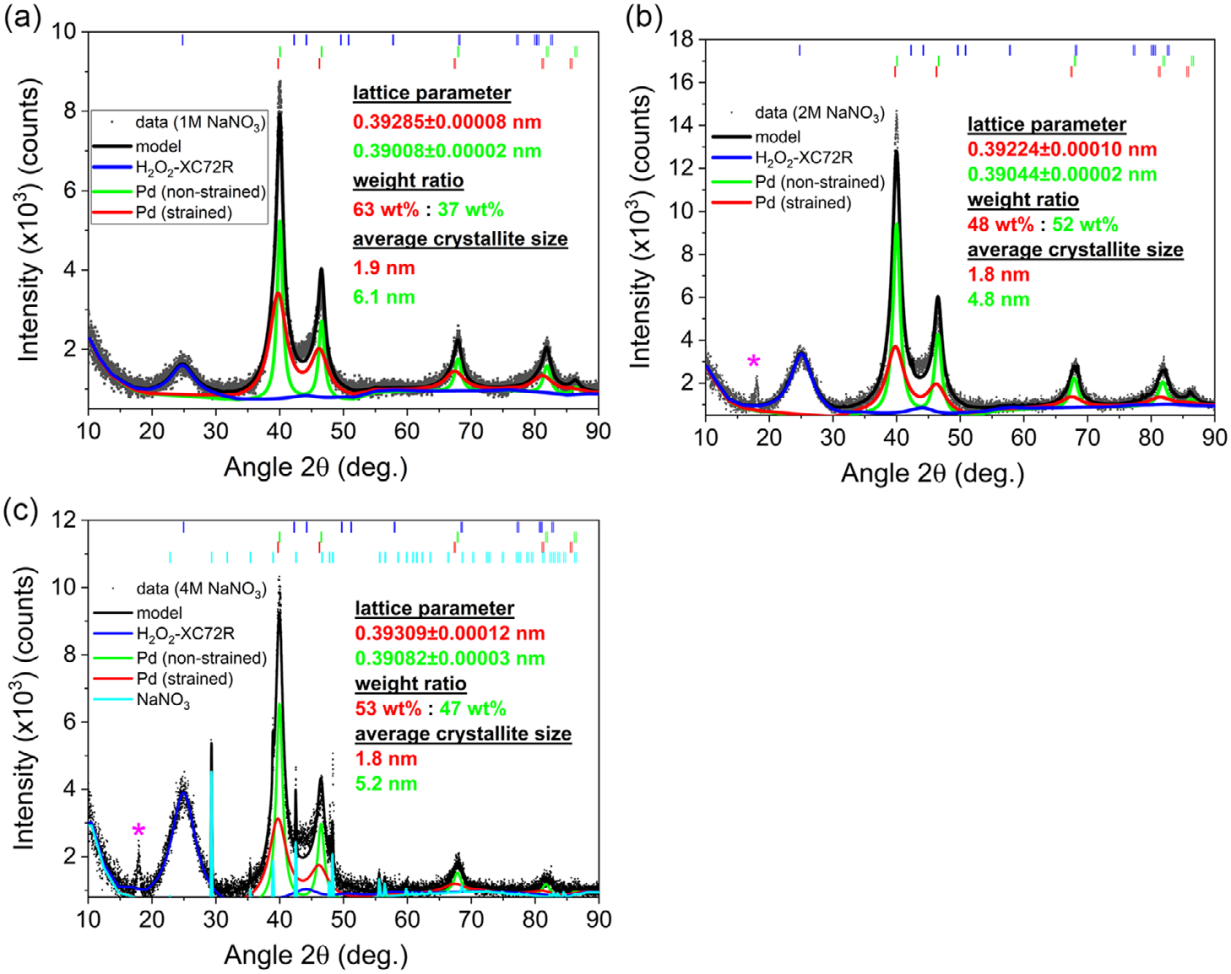
X‐ray diffraction (XRD) patterns and Rietveld models of the Pd/C catalysts synthesized with ±25 V amplitude, 200 Hz sinusoidal potential signal in a) 1, b) 2, and c) 4 м NaNO_3_ (black curve). The blue curve represents the fit of the H_2_O_2_‐pretreated Vulcan carbon in the pattern. The red and green sub‐peaks correspond to the fit of the strained and non‐strained Pd, respectively. The cyan sub‐peaks can be attributed to the fit of NaNO_3_ residues in the Pd/C catalyst. Purple * correspond to an unidentified reflection.

In the following section, we examine the effect of NaNO_3_ concentration on the synthesized Pd NPs using X‐ray diffraction (XRD) and electrochemical characterization. We produced three batches of Pd/C catalyst in 1, 2, and 4 м NaNO_3_ by applying a ± 25 V, 200 Hz sinusoidal potential signal. **Figure** **4**a–c displays the recorded diffraction patterns of the Pd/C catalysts fabricated in 1, 2, and 4 м NaNO_3_, including the corresponding Bragg peak positions. Experimental diffraction profiles attributed to the Pd signal possess a nontrivial shape, which can hardly be described by a simple pseudo‐Voigt profile. A closer look at the peak shape and its systematic broadening yields the model, which is based on two different Pd phases characterized by different weight fractions, crystallite size, and lattice parameters (colored in red and green) on top of the broad contribution from the H_2_O_2_‐pretreated Vulcan carbon XC72R in blue. It is hypothesized that two phases correspond to unstrained and strained Pd, where the strain could potentially originate from the harsh synthesis conditions of the Pd NPs. It must also be mentioned that the secondary (strained) Pd phase is present in non‐negligible amounts and needs to be properly accounted. Applying a 25 V potential amplitude generates gaseous oxygen and hydrogen excessively, consequently causing irreversible deformation and stress within the Pd crystal structure. Alternatively, the strain could arise during the cathodic polarization of the wires if extensive quantities of atomic hydrogen were irreversibly absorbed into the Pd crystal lattice. This assumption agrees with the work of Zhao et al. and Benck et al. who observed similar signal patterns, including sub‐peaks originating from hydride formation in the case of Pd‐based materials.^[^ ^41^, ^42^ ^]^ The signal from non‐strained (green) and strained (red) Pd NPs was modeled using the full‐profile Rietveld method. Additionally, extra peaks emerged in the diffraction patterns for the Pd NPs synthesized in 4 м NaNO_3_, which we attributed to NaNO_3_ residues in the corresponding batch. Following lattice parameters (0.39008 ± 0.00002, 0.39044 ± 0.00002, and 0.39082 ± 0.00003 nm) were detected for non‐strained Pd components produced in 1, 2, and 4 м NaNO_3_, respectively. Contributions from strained Pd were characterized by slightly higher lattice parameters (0.39285 ± 0.00008, 0.39224 ± 0.00010, and 0.39309 ±0.00012 nm for the corresponding PdH_x_ NPs). Following the relation between the lattice parameter and H:Pd ratio in the PdH_x_ β phase from Zhao et al.^[^^41]^ the obtained H:Pd ratios correspond to ≈0.17, ≈0.14, and ≈0.18 for the Pd catalysts synthesized in 1, 2, and 4 м NaNO_3_, respectively. In addition to this, the weight ratio disparities between the two Pd phases were explored as well. In the case of the Pd/C synthesized in 2 and 4 м NaNO_3_, the difference between strained and non‐strained Pd is negligible. The strained Pd phase constitutes 48 and 53 wt% of the total Pd content in the Pd/C catalyst fabricated in 2 and 4 м NaNO_3_. In contrast to those batches, the Pd/C catalyst synthesized in 1 м NaNO_3_ contains a larger proportion of strained Pd, correlating to roughly 63 wt%. Next to the weight ratio comparison of strained and non‐strained Pd, we assessed their average crystallite size for the respective catalyst batches.^[^ ^43^ ^]^ The crystallite sizes for strained and non‐strained Pd differ significantly in each batch but remain comparable across the different Pd/C batches. For the NPs synthesized in 1 м NaNO_3_, the average crystallite sizes for strained and non‐strained Pd correspond to ≈1.9 and ≈6.1 nm. The average crystallite sizes for the Pd/C batches synthesized in 2 and 4 м NaNO_3_, and a summary of all parameters extracted from XRD analysis, can be found in Table S1 and S2, Supporting Information.

### 2.4 Electrochemical Measurements

**Figure 5:**
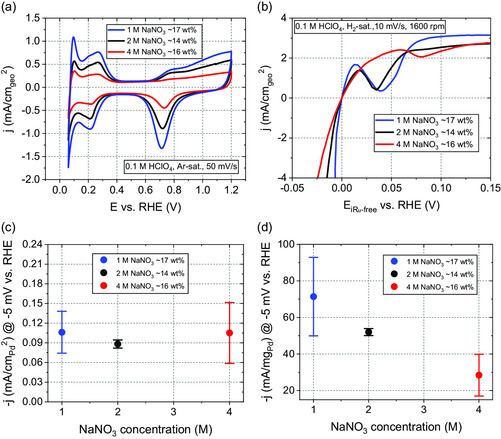
a) Cyclic voltammograms (CVs) of Pd/C catalysts synthesized in NaNO_3_ of various concentrations (1, 2, and 4 м). The curves were recorded in Ar‐saturated 0.1 м HClO_4_ at a scan rate of 50 mV s^−1^ and rotation speed of 400 rpm. b) Characteristic *iR*_u_‐corrected HER polarization curves (cathodic scan) of Pd/C catalysts, recorded in H_2_‐saturated 0.1 м HClO_4_ at a scan rate of 10 mV s^−1^ and a rotation speed of 1600 rpm. Comparison of the c) specific activity (*SA*) and d) mass activity (*MA*) evaluated at −5 mV versus RHE with the NaNO_3_ synthesis electrolyte concentration.

**Figure** **5**a displays the cyclic voltammograms (CVs) recorded in Ar‐saturated 0.1 м HClO_4_ for the synthesized samples in 1, 2, and 4 м NaNO_3_. Despite slight variations in mass loading, which are smaller than 3%, the intensity of the H absorption/adsorption and H desorption peaks in the CVs increase with decreasing NaNO_3_ concentration during synthesis. A similar tendency appears for the specific surface area (*SSA*), which increases almost linearly with a decrease in the electrolyte concentration. The lowest *SSA* of 27.1 ± 5.0 m^2^ g_Pd_^−1^ corresponds to the Pd/C catalyst fabricated in 4 м NaNO_3_. Accordingly, the *SSA* increases to 58.9 ± 3.5 and 67.3 ± 0.2 m^2^ g_Pd_^−1^ for the catalysts manufactured in 2 and 1 м NaNO_3_, respectively.

In our catalytic system, the *SSA* primarily varies with the size and shape of Pd NPs and their state of aggregation and distribution. Nevertheless, a larger *SSA* could modify the activity of the Pd/C catalyst for surface‐sensitive reactions like the HER. This theoretical hypothesis coincides with our observations from the polarization curves conducted in H_2_‐saturated 0.1 м HClO_4_, as displayed in Figure 5b. In the cathodic scan of the polarization curve, a characteristic peak occurs at slightly positive potentials, which is typical for Pd and relies on the formation of PdH_x_.^[^^44]^ The geometric activity (*GA*) evaluated at −5 mV versus the reversible hydrogen electrode (RHE) increases for the NPs prepared with decreasing NaNO_3_ concentrations, starting with 0.44 ± 0.18, 0.75 ± 0.03, and ultimately reaching 1.23 ± 0.37 mA cm_geo_^−2^ for the Pd/C catalysts synthesized in 4, 2, and 1 м NaNO_3_, respectively.

In addition to the *GA* evaluation, we investigate trends of the mass activity (*MA*) and specific activity (*SA*) in correlation with the used NaNO_3_ synthesis electrolyte concentration of the Pd/C catalysts. Figure 5c relates the NaNO_3_ synthesis concentration to the evaluated *SA*. For the Pd/C samples synthesized in 1, 2, and 4 м NaNO_3_, the *SA* evaluated at −5 mV versus RHE corresponds to 0.11 ± 0.03, 0.09 ± 0.01, and 0.11 ± 0.05 mA cm_Pd_^−2^, respectively. Schmidt et al. correlated the strain in the crystal structure from subsurface hydride layers to the change in adsorption Gibbs free energy (*ΔG*) of the hydrogen intermediate for Pd.^[^ ^37^ ^]^ As a result, *ΔG* and, thus, the intrinsic HER activity depend on the number of subsurface hydride layers and the H:Pd ratio. Under consideration of the error bars of the *SA* reported in Figure 5c, its values match remarkably well for the catalysts synthesized in 1, 2, and 4 м NaNO_3_, which coincides with the comparable H:Pd ratio for the three catalysts mentioned earlier. Figure 5d depicts the dependence of the *MA* on the NaNO_3_ synthesis electrolyte concentration. The *MAs* evaluated at −5 mV versus RHE correspond to 71.4 ± 21.5, 52.0 ± 1.9, and 28.4 ± 11.4 mA mg_Pd_^−1^ for the Pd/C catalysts synthesized in 1, 2, and 4 м NaNO_3_, respectively. The enhanced *MA* of the Pd/C catalyst synthesized in 1 м NaNO_3_ can most likely be attributed to the larger *SSA* and the enhanced amount of strained Pd NPs of ≈63 wt% compared to non‐strained Pd NPs. This electrochemical characterization underlines the effect of the NaNO_3_ synthesis concentration on the resulting *SSA* and HER activity.

**Figure 6:**
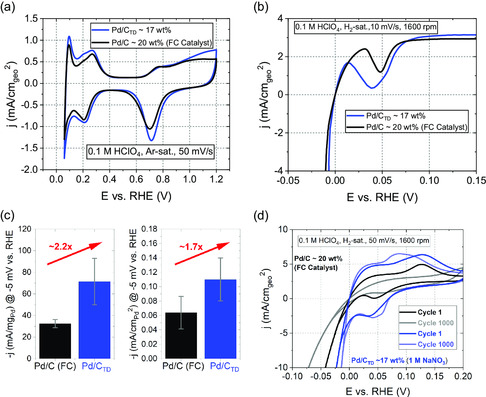
a) CVs of the commercial Pd/C FC catalyst and the Pd/C_TD_ catalyst synthesized in 1 м NaNO_3_ by electrochemical erosion. The curves were recorded in Ar‐saturated 0.1 м HClO_4_ at a scan rate of 50 mV s^−1^ and rotation speed of 400 rpm. b) Characteristic *iR*_u_‐corrected HER polarization curves (cathodic scan) of both catalysts, recorded in H_2_‐saturated 0.1 м HClO_4_ at a scan rate of 10 mV s^−1^ and a rotation speed of 1600 rpm. c) Comparison of the *MA* and *SA* evaluated at –5 mV versus RHE of both catalysts. d) Durability test by conducting 1000 HER cycles for both catalysts. The characteristric *iR*_u_‐corrected HER polarization curves (cathodic and anodic scan) were recorded in H_2_‐saturated 0.1 м HClO_4_ at a scan rate of 50 mV s^−1^ and rotation speed of 1600 rpm.

As a last step, we compared the electrochemical performance of our most active catalyst toward HER with a commercial 20 wt% Pd/C catalyst (brand: Fuel Cell Store (FC catalyst)). For the FC catalyst, the Pd NPs are supported on Vulcan XC‐72 and exhibit a crystallite size of 3–5 nm.^[^ ^45^ ^]^ The crystallite size can be roughly treated as an approximation of the size of the NPs, which corresponds to our catalyst (Table S2, Supporting Information), synthesized using the electrochemical erosion, TD approach in 1 м NaNO_3_. For clarity, we will refer to our synthesized catalyst as Pd/C_TD_ in the following section. As noted in the last paragraph, the *SSA* of the Pd/C_TD_ catalyst corresponds to ≈67.3 ± 0.2 m^2^ g_Pd_^−1^, which is ≈1.3 times greater than the *SSA* of the commercial FC catalyst, measured as 50.9 ± 1.8 m^2^ g_Pd_^−1^. This difference in the *SSA* is apparent in the recorded CV profiles of both catalysts, displayed in **Figure** **6**a. Across all peaks arising from faradaic processes, the peaks of the TD synthesized Pd/C_TD_ demonstrate higher currents. Regarding the HER activity, the Pd/C_TD_ catalyst outperforms the commercial FC catalyst, as shown in the cathodic scans of the polarization curves (Figure 6b). The *GA* evaluated at −5 mV versus RHE corresponds to 0.69 ± 0.09 and 1.23 ± 0.37 mA cm_geo_^−2^ for the commercial Pd/C and Pd/C_TD_ catalyst, respectively. As shown in Figure 6c, the *MA* of the Pd/C_TD_ corresponds to 71.4 ± 21.5 mA mg_Pd_^−1^ and is, therefore, ≈2.2 times larger than the *MA* of 32.5 ± 3.81 mA mg_Pd_^−1^ of the Pd/C FC catalyst. The *SA* follows a similar trend, with Pd/C_TD_ revealing an activity of 0.11 ± 0.03 mA cm_Pd_^−2^ and thus surpassing the *SA* of 0.06 ± 0.02 mA cm_Pd_^−2^ of the commercial FC catalyst by 1.7 times. As discussed earlier, among other influences related to differences in size and shape of the Pd NPs, the intrinsic HER activity depends on the number of subsurface hydride layers and the H:Pd ratio of the Pd catalyst.^[^ ^37^ ^]^ Due to the electrochemical erosion synthesis, strain emerges within the crystal structure of the Pd/C_TD_, which could significantly promote the formation of PdH_x_ under reaction conditions, in contrast to the commercial Pd/C FC catalyst. This is evidenced by a distinct peak occurring at a slightly positive potential in the cathodic scan of the catalyst, indicating hydrogen absorption, which is undeniably more pronounced and broader for the Pd/C_TD_ catalyst and could explain its higher activity due to PdH_x_ formation.^[^ ^37^ ^]^ In addition, durability tests of the two investigated catalysts were conducted by executing 1000 HER cycles for both catalysts, as displayed in Figure 6d. Over cycling, the commercial Pd/C catalyst demonstrated a progressive decline in current of the polarization curve, most likely due to the dissolution of Pd NPs, a known phenomenon for Pd NPs in HClO_4_ electrolyte.^[^ ^46^ ^]^ In contrast, the Pd/C_TD_ catalyst exhibited a current increase over the same number of cycles. To explain this difference between the two catalysts, we need to consider the peak occurring at slightly positive potentials in the cathodic scan once more, which is related to PdH_x_ formation. For the commercial Pd/C FC catalyst, this absorption peak almost completely vanishes over 1000 cycles, suggesting that hydrogen absorption by the crystal structure no longer occurs as the Pd NPs reach their maximal accessible H:Pd ratio. Conversely, for the Pd/C_TD_ NPs, the peak broadens over 1000 cycles, indicating that even more hydrogen is absorbed compared to the first cycle. This continual absorption of hydrogen is crucial to understanding the increase in HER activity since an increased H:Pd ratio and an expanded amount of Pd subsurface layers containing hydrogen enhance the HER activity, as mentioned earlier.^[^ ^37^ ^]^ Furthermore, we mention that a part of the improvement could also be attributed to the continual reduction of PdO (30% in Pd/C_TD_ as determined via the XPS analysis in Figure 3) under reducing potentials during cycling.

## 3 Conclusion

The synthesis of Pd nanostructures using electrochemical erosion without surfactants was not reported in the literature so far since the organic additives hinder the significant agglomeration processes for Pd. In our work, we developed a procedure based on the pretreatment method of Pd wires before electrochemical erosion and the choice of a suitable nontoxic electrolyte (NaNO_3_) that allowed us to synthesize homogenously distributed, non‐agglomerated NPs supported on Vulcan carbon. Furthermore, we discovered that the concentration of NaNO_3_ in the synthesis suspension significantly affects the properties of the formed NPs. Using 1 м NaNO_3_ leads to the formation of the most efficient NPs with a mean diameter of 6.4 ± 2.9 nm and an *SSA* of 67.3 ± 0.2 m^2^ g_Pd_^−1^. Electrochemical characterization toward the HER revealed a significant *MA* of 71.4 ± 21.5 mA mg_Pd_^−1^ evaluated at −5 mV versus RHE, which we attributed to an enhanced amount of strained Pd NPs of ≈63 wt% compared to non‐strained ones. In addition, we compared the activity and stability between the Pd/C catalyst synthesized in 1 м NaNO_3_ and a commercially available Pd/C catalyst. The results demonstrate that the Pd/C catalyst produced through electrochemical erosion outperforms the commercial catalyst in terms of *MA* and *SA* for the HER, exhibiting a ≈2.2‐ and ≈1.7‐fold enhancement, respectively. During the stability tests, the activity of the commercial catalyst gradually declined, while the Pd/C catalyst synthesized via electrochemical erosion exhibited an increase in activity. This enhancement can be attributed to a continuous hydride formation process, effectively increasing the HER activity.

## 4 Experimental Section

4.1

4.1.1

##### Vulcan Carbon XC72R Pretreatment

Despite working with aqueous electrolytes, we selected Vulcan carbon XC72R as a support material. The oxidation of the Vulcan carbon surface in an H_2_O_2_ pretreatment step improved hydrophilicity and wettability.^[^ ^40^ ^]^ For the pretreatment, Vulcan XC72R (1 g) was dispersed in 30% H_2_O_2_ (100 mL, 30% H_2_O_2_, p.a., ISO, Carl Roth, Germany) and continuously stirred for ≈12 h at ≈70 °C. Afterward, the stirred suspension was diluted with ultrapure H_2_O (18.2 MΩcm, Merck Millipore, USA), followed by a filtering and an H_2_O‐washing step. The removal of water residues in the powder was accomplished by drying it at 60 °C for ≈16 h.

##### Electrochemical Erosion

Electrochemical erosion of bulk Pd wires (Ø = 0.25 mm, 99.95%, MaTecK GmbH, Germany) generated active NPs, if the bulk wires underwent a pretreatment step to modify their properties beforehand. In our work, these modifications consisted of an HER pretreatment, followed by an annealing pretreatment of the Pd wires. The HER step involved cyclic voltammetry conducted with the Pd wires in a three‐electrode cell filled with 0.1 м HClO_4_ (70% HClO_4_, extra pure, Acros, Germany). The electrolyte was purged for 30 min with H_2_ gas (5.0, Westfalen, Germany) to avoid undesired gaseous compounds and ensure stable equilibrium potential conditions. Further, 100 potential cycles with a scan rate of 50 mV s^−1^ were executed with vertex potentials of 0.82 and −0.38 V versus RHE. Potentials lower than 0 V versus RHE evolved molecular hydrogen, which partially absorbed and therefore induced stress in the Pd crystal structure. Subsequently, these hydrogen‐containing Pd wires were annealed in an inductive heater (20–80 kHz, 15 kW, SP‐15 A, MTI, USA). Inside the heater, an Ar (5.0, Westfalen, Germany) atmosphere hindered Pd–oxide formation during annealing up to 900–1050 °C for five cycles of 90 s. Between instances, 5 min breaks with persistent Ar flow guaranteed rapid cooling of the wires. During annealing, the absorbed hydrogen escaped from the Pd wires, leading to defects in the Pd bulk and surface. In addition, structural changes arose during the rapid cooling afterward, which was discussed in the results section. To increase the density of structural defects, we repeated the HER and annealing pretreatment either once or thrice for each wire. During electrochemical erosion, a potential was applied to Pd wires immersed in a suspension to produce NPs at their surface. The suspensions consisted of H_2_O_2_‐pretreated Vulcan XC72R (≈20 mg) dispersed in a NaNO_3_ (ACS, ISO, Reag. Ph Eur, Merck, Germany) electrolyte solution. Further, ethanol (5 mL, EMSURE® ACS, ISO, Reag. Ph Eur, Sigma Aldrich®, USA) was added to enhance the Vulcan carbon's hydrophilic character and ensure its excellent dispersion next to ultrasonication treatments. Parameters like the NaNO_3_ concentration of the suspension affected the NP formation process. Accordingly, by applying a ± 25 V, 200 Hz sinusoidal AC voltage profile to Pd wires, NPs were synthesized in suspensions with NaNO_3_ concentrations of 1, 2, and 4м under continuous stirring of ≈500 rpm. The effect of frequency variations was investigated in 1 м NaNO_3_ suspensions applying a ± 25 V, sinusoidal AC voltage profile with 20, 100, or 200 Hz. Comparable mass loadings of the different samples arose by continuous erosion, until the mass of the initial Pd wires diminished by ≈5 mg. This mass of synthesized Pd NPs resulted in a 20 wt% theoretical mass loading of the Pd/C catalyst since the synthesis suspensions consisted of ≈20 mg H_2_O_2_‐pretreated Vulcan XC72R. After the synthesis, the mixture was stirred at 500 rpm for 16 h. Subsequently, the suspension was filtered in a Büchner funnel, washed with a mixture of ultrapure water and ethanol, and dried in a furnace at 60 °C for 12 h.

##### Catalyst Inks

Several characterization methods relied on catalyst inks produced with the catalyst powder. These inks consisted of Pd/C (10 mg), ultrapure H_2_O (3600 μl), isopropanol (1446 μl, puriss. p.a., ACS reagent, ≥ 99.8%, Sigma Aldrich®, USA), and Nafion dispersion (30 μl, 5 wt% in lower aliphatic alcohols and water, Sigma Aldrich, USA).

Consecutively, a 5 min ultrasonication treatment ensured a homogeneous distribution of the nanostructured catalyst.

##### Thermogravimetric Analysis

For thermogravimetric analysis (TGA), a Mettler Toledo TGA instrument determined the Pd to carbon support weight ratio of the Pd/C catalyst powder by recording the sample's mass versus time for a predefined temperature profile. The profile consisted of three main steps: removal of residual water at 135 °C in Ar, carbon oxidation at 800 °C in O_2_ with simultaneous Pd oxide formation, and Pd oxide reduction at 800 °C in Ar. For the steps at 800 °C, the temperature was held constant for 30 min per step. The heating speed and gas airflow corresponded to 50 K min^−1^ and 50 mL min^−1^, respectively.

##### Characterization

Powder XRD provided the crystallographic and structural properties of the synthesized Pd/C‐nanostructured catalyst. For this purpose, the samples were illuminated with a Cu–Kα (λ = 1.5406 Å) source with an integrated Ni‐based filter by a Rigaku MiniFlex 600‐C. The device recorded diffraction patterns in a slow‐scanning mode with a (5° min^−1^) step velocity from 5 to 90°. Data analysis was performed applying full‐profile Rietveld method as implemented in the program FullProf.^[^ ^47^ ^]^ Contribution to diffraction patterns were modeled assuming isotropic size distribution, instrumental resolution was determined from Si reference measurements.

The TEM analysis was used for the Pd/C catalyst synthesized in 1 м NaNO_3_ and ±25 V, 200 Hz sinusoidal AC voltage signal to visualize the morphology and chemical composition of the synthesized nanostructure in high resolution. The TEM was performed with an image spherical aberration corrected TITAN Themis 60‐300 (Thermo Fischer Scientific, USA) microscope operated at 300 keV. The analysis was conducted via HRTEM imaging with spherical aberration corrected to ≈0 μm, STEM imaging with a high‐angle annular dark‐field detector, and STEM–EDX elemental mapping with SUPER‐X spectrometer. The STEM–EDX maps of C–K, O–K, and Pd–L were created by quantification of the collected shell signal of the elements using Cliff–Lorimer k factors^[^ ^48^ ^]^ to weight percentage. All TEM data acquisition and processing were done with a software Velox v. 2.14. The size of the NPs was estimated using the software ImageJ.^[^ ^49^ ^]^ In addition, the Pd NPs synthesized with 1 м NaNO_3_ and ±25 V AC voltage signal with 200, 100, and 20 Hz were visualized by TEM. It was performed using a Jeol 2010 (CMTC–INPG) microscope with a LaB_6_ filament and operating at 200 kV, with a 0.19 nm point to point resolution. The images were collected with a 2018 × 2048 pixels charge‐coupled device (CCD) camera (Gatan Ultrascan 1000 XP).

XPS elucidated the catalysts’ elemental composition and chemical state, including content estimations. The XPS device consisted of a SPECS XR 50 (SPECS, Germany) X‐ray source with a non‐monochromatized Al–Kα anode (1486.61 eV) and a PHOIBOS 150 hemispherical energy analyzer (SPECS, Germany) with a 150 mm mean radius. All spectra were acquired in an ultrahigh vacuum chamber at an operating pressure below 5 × 10^−9^ mbar. The recorded XPS data was evaluated using the Casa XPS software (Version 2.3.25rev1.1 J).

##### Electrochemical Measurements

Pd/C catalysts were electrochemically characterized in a three‐electrode setup consisting of the reference (RE), the counter (CE), and the working electrode (WE). Those electrodes were immersed in 0.1 м HClO_4_ electrolyte within a glass cell previously cleaned with Caro's acid/boiling water. This prevailing cleaning procedure consisted of a 3:1 mixture of sulfuric acid (96% H_2_SO_4_ Suprapur, Merck, Germany) and H_2_O_2_. The acidic mixture remained in the glass cells for ≈12 h. Following that, the glassware was washed several times with boiled ultrapure water.

A mercury/mercurous sulfate electrode (Si Analytics, Germany) was used as an RE in our experiments. The CE consisted of a multiple‐circled Pt wire, predominantly owing to its high electrical conductivity, mechanical robustness, and excellent electrocatalytic activity.^[^ ^50^ ^]^ The WE included a rotating disc electrode consisting of an OrigaTrod electrode rotator (OrigaLys ElectroChem SAS, France) and a suitable electrode tip. The tip comprised a 5 mm glassy carbon disk shielded by a polyether ether ketone coverage, hindering the exposure of glassy carbon edges or side surfaces. This configuration guaranteed a well‐defined geometric glassy carbon surface (0.196 cm^2^) on which the aforementioned electrocatalyst inks (10 μL) were drop‐casted. Before ink deposition, the glassy carbon tips were polished with 1.0, 0.3, and 0.05 μm grain‐sized alumina paste (MicroPolishTM, Buehler, USA). After ink deposition, the hot air of a heat gun (D5950 Remington, Germany) dried the ink under persistent rotation.

The synthesized Pd/C catalysts were electrochemically characterized by applying different CV techniques. Those included voltammetry in Ar‐ and H_2_‐saturated electrolytes. For this, we purged the 0.1 м HClO_4_ electrolyte with Ar for 30 min to ensure the absence of O_2_. Subsequently, we executed the measurement at a scan rate of 50 mV s^−1^ and a rotation speed of 400 rpm until reaching a steady state in the potential window between 0.06 and 1.2 V versus RHE.

Further, to determine the electrochemically active surface area (*ECSA)*, the CO‐stripping method was used. For this method, purging 0.1 м HClO_4_ with CO (1000 ppm CO in Ar, 4.7/5.0, Westfalen, Germany) for 15 min secured a CO‐saturated electrolyte. Due to the lengthy purging procedure with CO, applying 0.1 V versus RHE for 50 min under 400 rpm formed a CO monolayer with maximal coverage on the catalyst surface. Subsequently, the electrolyte was purged with Ar for 15 min before two CV cycles were executed with a scan speed of 10 mV s^−1^ without rotation in the potential window between 0.06 and 1.2 V versus RHE. The CO‐stripping experiment defined the CO oxidation charge, which needed to be divided by the adsorbed CO surface coverage and the oxidation charge of a CO monolayer per unit area.^[^ ^51^ ^]^ Both parameters significantly depended on the crystal orientation,^[^ ^52^, ^53^ ^]^ thus making the situation difficult for polycrystalline NPs. With the simplification in mind, we followed other reports and assumed a CO surface coverage of 1 and a CO desorption per unit area of ≈420 μC cm_Pd_^−1^.^[^ ^54^ ^]^ Furthermore, the *ECSA* was normalized by the determined Pd mass of the ink coatings deposited on the glassy carbon. This normalization resulted in the *SSA* and was essential for nanostructured electrocatalysts with different mass loadings.

For the HER/HOR measurements, the 0.1 м HClO_4_ electrolyte was continuously purged with H_2_ for 30 min to saturate the electrolyte. The H_2_‐saturation guaranteed stable equilibrium potential during the cycling with a scan rate of 10 mV s^−1^ and a rotation speed of 1600 rpm in the potential window from 0.82 to −0.13 V versus RHE. Since negative RHE potentials evolved hydrogen, we evaluated the HER activity at −5 mV versus RHE from the cathodic polarization curve, which was additionally corrected for the scan rate. Furthermore, the measured potentials of the polarization curves were *iR*_u_ corrected with the help of electrochemical impedance spectroscopy (EIS). The uncompensated resistance was determined by frequency perturbation of the applied potential in the 100 kHz to 10 Hz range with a 25 mV perturbation amplitude at different potentials. The EIS measurements were conducted in Ar‐saturated 0.1 м HClO_4_ under rotation speed of 1600 rpm.

Furthermore, the HER activities, evaluated at −5 mV versus RHE, were presented as *SA* and *MA*. For the former, the measured current was normalized by the *ECSA* obtained from the CO‐stripping measurement. For the latter, the current was divided by the mass of the Pd NPs present in the ink coating on the glassy carbon.

For the durability test consisting of 1000 HER cycles, the 0.1 м HClO_4_ electrolyte was continuously purged with H_2_ for 30 min. The CVs were executed within a potential window from 0.22 to −0.13 V versus RHE with a scan rate of 50 mV s^−1^ and rotation speed of 1600 rpm.

## Conflict of Interest

The authors declare no conflict of interest.

## Supporting information

Supplementary Material

## Data Availability

The data that support the findings of this study are available from the corresponding author upon reasonable request.
