# Supplementary material for: Top‐down Surfactant‐Free Synthesis of Supported Palladium‐Nanostructured Catalysts
Source: Small Sci. 2024 Jan 11;4(3):2300241. doi: 10.1002/smsc.202300241 (PMC11935149; doi:10.1002/smsc.202300241)
Supplement: Supplementary file 1 — Supplementary Material [file SMSC-4-2300241-s001.pdf]

Supporting Information

Top-Down Surfactant-Free Synthesis of Supported Palladium Nanostructured Catalysts

Christian M. Schott, Peter M. Schneider, Kais Sadraoui, Kun-Ting Song, Batyr Garlyyev, Sebastian A. Watzele, Jan Michalička, Jan M. Macak, Arnaud Viola, Frédéric Maillard, Anatoliy Senyshyn, Johannes A. Fischer, Aliaksandr S. Bandarenka, Elena L. Gubanova**

To synthesize Pd nanoparticles (NPs), we applied a ±25 V sinusoidal AC potential signal to bulk Pd wires immersed in a suspension containing an electrolyte and the carbon support. The choice of an appropriate electrolyte is mandatory since the respective ions drastically affect the erosion of bulk wires. The technique demonstrates various methodological parameters, like electrolyte concentration and the frequency of the sinusoidal AC potential. Both parameters directly influence the properties of the synthesized Pd NPs.

The most elaborated NP formation theory attributes the critical role in this process to the cation of the electrolyte.[i] Nevertheless, in the case of Pd, we found that the anion significantly influences the electrochemical erosion process. Under application of a ±25 V, 200 Hz sinusoidal potential signal, erosion failed in 1 ᴍ sodium carbonate (Na_2CO_3), acetate ($C_2H_3NaO_2$), perchlorate ($NaClO_4$), and sulfate (Na_2SO_4); but succeeded in sodium nitrite ($NaNO_2$) and nitrate ($NaNO_3$).

For $NaNO_2$, the formed NPs rapidly agglomerated after production, prohibiting any catalytic application. In the case of $NaNO_3$, we did not detect notable particle agglomeration after erosion. Nevertheless, we highlight that the wires in $NaNO_3$ only erode if they already exhibit a rough surface containing numerous defects. The roughness of the wires can result from various pretreatment strategies. Hence, we invented a wire pretreatment process, which tremendously improved the erosional behavior of Pd wires for the reproducible synthesis of Pd NPs in a $NaNO_3$ electrolyte. SEM experiments clarified the effect of the two-step pretreatment by comparing the morphology of the Pd wire before and after the process, as illustrated in **Figure S1a** and **S1b**, respectively. Initially, the pristine surface exposes multiple deep grooves and cracks

next to flat areas with significant size. After pretreatment, the flat areas disappeared, and simultaneously, the amount of visible deep grooves or cracks drastically increased. Additionally, slightly larger surface areas appear rough, presumably emerging from multiple nano-grooves or cracks that cannot be visualized due to the limited resolution of the SEM. Furthermore, **Figure S1c** magnifies the surface after the pretreatment and indicates that the grooves or cracks develop at grain boundaries. We assume that erosion occurs at those grooves at the grain boundary due to the large defect density in this distorted region. Additional SEM measurements after erosion validate this hypothesis since the grooves at grain boundaries significantly enlarge during the erosion, as depicted in **Figure S1d**. Due to the large grooves, plateaus are visible on the entire surface, as shown in the inset of Figure S1d, which correspond to the initial grains of the Pd wire.

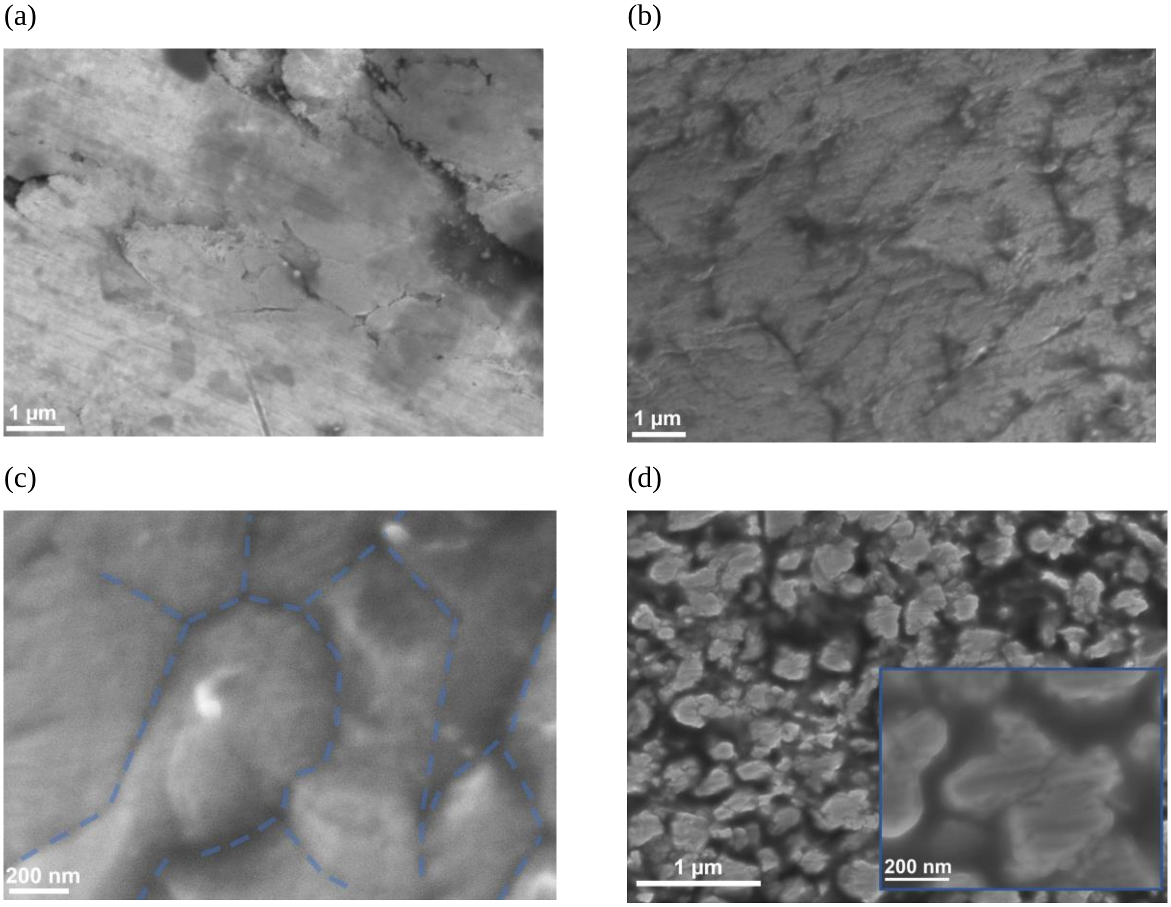

Figure S1. SEM images of (a) a pristine Pd wire and (b) the same wire after the pretreatment procedure. (c) Magnification of (b) to illustrate that the Pd wire cracks at the grain boundaries. (d) SEM image of the pretreated wire after the erosion with a ±25 V, 200 Hz sinusoidal potential signal in 1 ᴍ $NaNO_3$. The inset magnifies the surface (d) after erosion.

Alternatively, to variation in the $NaNO_3$ concentration, we investigated the effect of the frequency on Pd NP formation. Therefore, three batches in 1 ᴍ $NaNO_3$ were fabricated by applying a ±25 V sinusoidal potential signal with 200 Hz, 100 Hz, and 20 Hz frequency. As highlighted in the experimental part, the Pd wires for this synthesis were only treated once by the HER and annealing procedure. For morphological information, we executed TEM characterization of the Pd/C catalysts fabricated with 200 Hz, 100 Hz, and 20 Hz frequency, shown in **Figure S2a-S2d**. Two different types of particles coexist in all three elucidated samples. Firstly, small individual particles can be found, for which we estimate a diameter of ~7-8 nm. Secondly, large NPs with a diameter of roughly 30 nm are detected. For all samples, we created size

distribution histograms distinguishing between the small and bigger NPs, as shown in **Figure S3a-S3c**. For the bigger Pd NPs synthesized with 200 Hz, 100 Hz, or 20 Hz frequency, the size corresponds to 31±11 nm, 25±8 nm, and 33±11 nm, respectively. For the smaller Pd NPs, the size changes even less noticeably from 7.0±2.0 nm to 8.0±2.0 nm to 8.0±3.0 nm, respectively. Besides the Pd NP size, the mass activity (*MA*) depends on the NP's shape and the defect density or roughness factor. Regarding the shape of the NPs, the recorded TEM images again reveal two different types of particles, which can explain the dissimilar *MA* reported above. For the Pd/C catalyst synthesized with 200 Hz frequency (Figure S2a**)**, the Pd NPs display smooth surfaces without observable defect states. However, for the Pd/C catalyst produced with 100 Hz and 20 Hz frequencies (Figure S2b and S2c), the particles emerge in a "cloudy" formation without smooth surfaces of significant size. The surface appears scattered, indicating a large density of defect states.

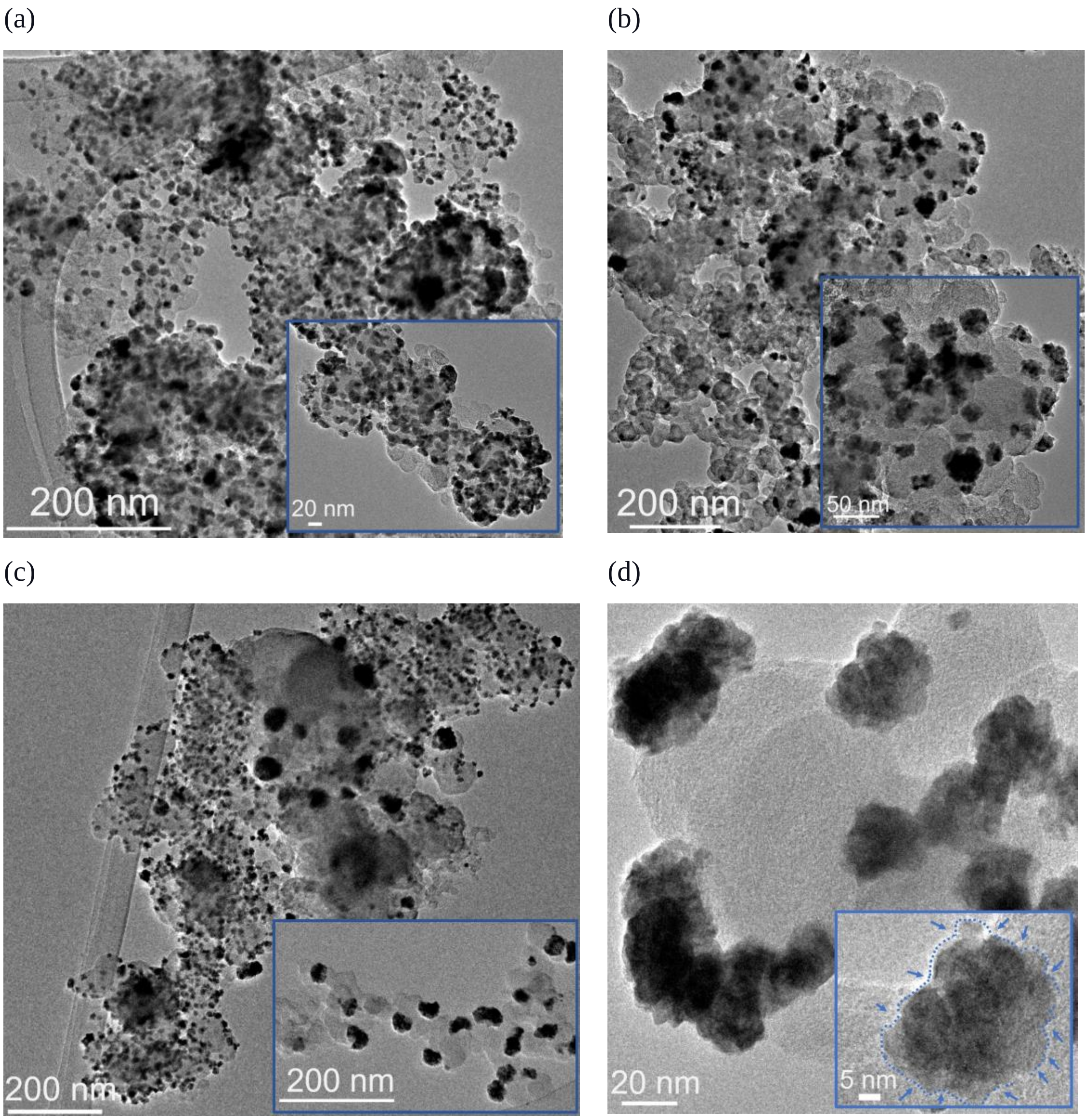

Figure S2. TEM images of the Pd/C catalysts synthesized with (a) 200 Hz, (b) 100 Hz, and (c)-(d) 20 Hz frequency with 200 nm and 20 nm resolution scale bars, respectively. The inset in (d) shows a single particle with a 5 nm resolution scale bar. The dashed line indicates the "cloudy" shape of the NP. The blue arrows point to concave-like defects.

(a) (b)

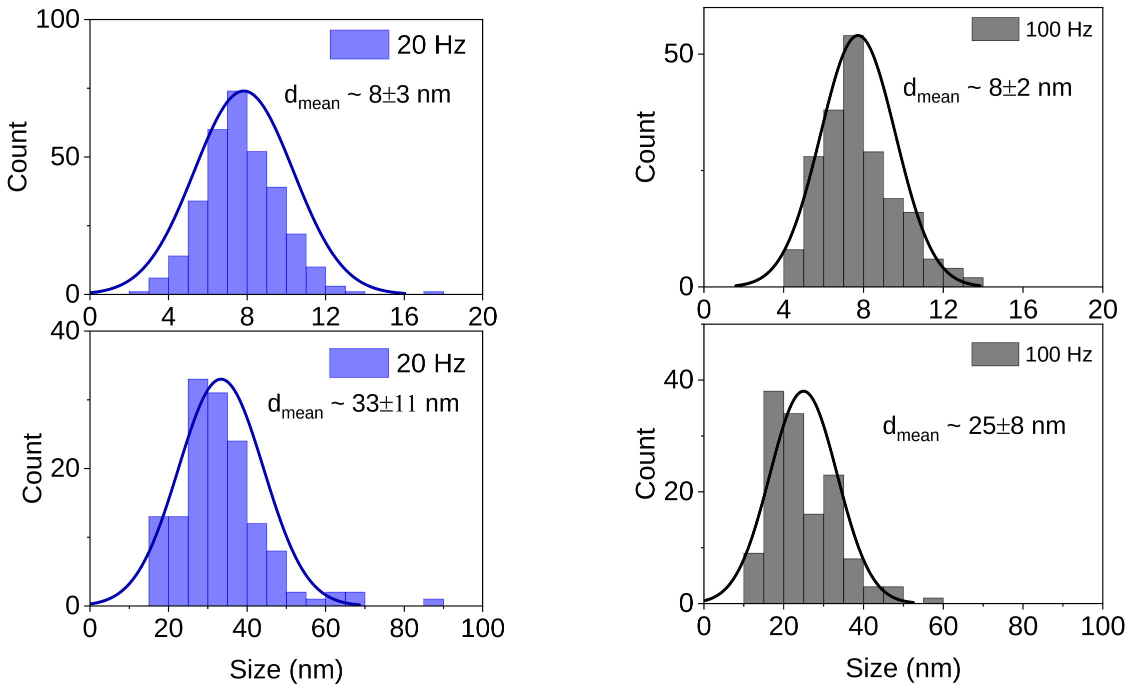

20 Hz
dmean ~ 8±3 nm
Count
dmean ~ 33±11 nm
Size (nm)
100 Hz
dmean ~ 8±2 nm
dmean ~ 25±8 nm

(c)

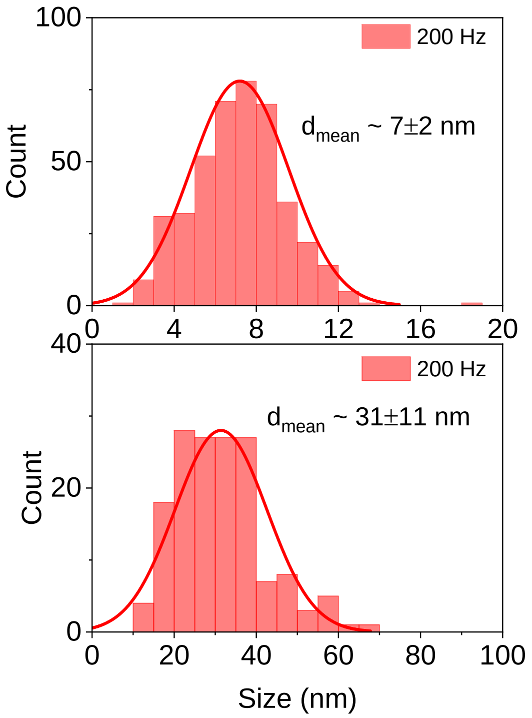

Figure S3. Distribution of the Pd NPs synthesized with (a) 20 Hz (b) 100 Hz and (c) 200 Hz frequency in 1 M $NaNO_3$ and one pretreatment before erosion.

Figure S4a-S4c provide the collected diffraction patterns of the Pd/C catalysts manufactured with 200 Hz, 100 Hz, and 20 Hz, respectively. In line with the previously investigated catalysts, the XRD patterns reveal the presence of two Pd phases attributed to strained and non-strained Pd for the Pd/C catalysts. The lattice parameter of the strained Pd NPs corresponds to approximately 0.39950±0.00010 nm, 0.40107±0.00006 nm, and 0.40025±0.00006 nm for an applied frequency of 200 Hz, 100 Hz, and 20 Hz, respectively. Assuming the strain arises from hydride formation, the H:Pd ratios reach ~0.43, ~0.49, and ~0.46, respectively.[ii] Similar to the previously investigated catalyst, we explored the weight ratio differences of the strained and non-strained Pd phases for the catalysts synthesized with different applied frequencies. Furthermore, independently of the strain presence, the frequency does not affect the crystallite size of the Pd NPs. An overview of all parameters extracted from XRD experiments can be found in **Table S1, and S2**.

(a) (b)

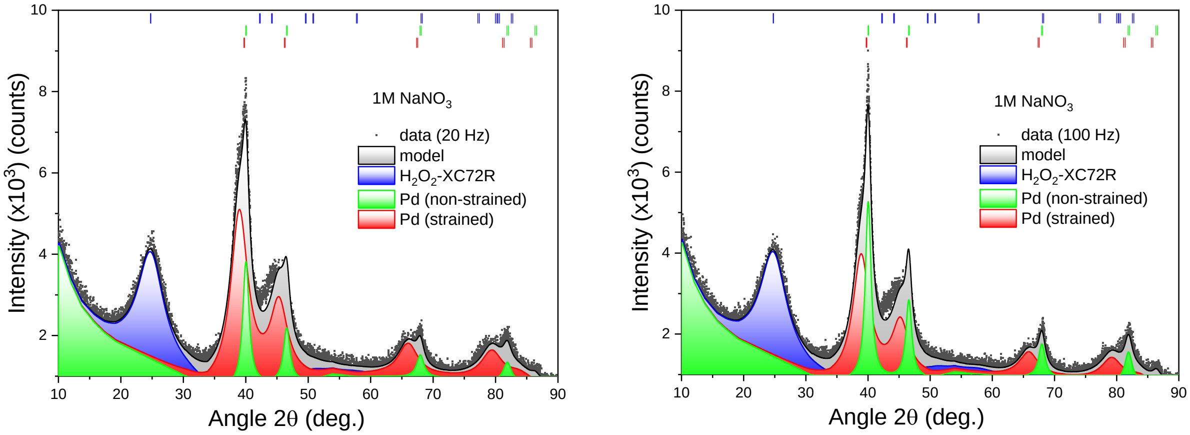

(c)

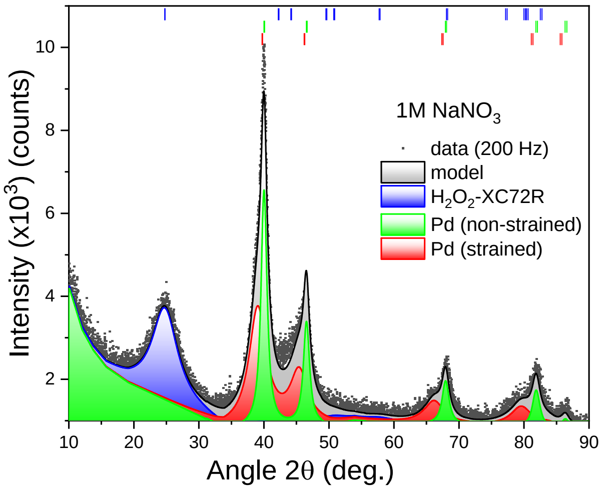

Figure S4. XRD patterns of the Pd/C catalyst synthesized with different applied frequencies in 1 M $NaNO_3$ with one pretreatment before erosion.

Figure S5a-S5d display the cyclic voltammograms recorded in Ar-saturated 0.1 M $HClO_4$ for the Pd/C catalysts produced with 200 Hz, 100 Hz, and 20 Hz frequencies, respectively. The small mass loading dissimilarities make the use of geometrical current densities reasonable to compare the size of characteristic Pd peaks. Those negligibly vary for a 2-fold frequency decrease from 200 Hz to 100 Hz but significantly change for the 10-fold frequency modification from 200 Hz to 20 Hz, as shown in Figure S5a. A similar trend arises for the specific surface areas (*SSA*), which continuously improves with a decrease in applied frequency. The lowest *SSA* of 36.4±0.7 $m^2\ g_{Pd}^{-1}$ corresponds to the Pd/C catalysts synthesized with 200 Hz frequency. Accordingly, the *SSA* increases to 40.8±2.3 $m^2\ g_{Pd}^{-1}$ and 46.5±4.9 $m^2\ g_{Pd}^{-1}$ for the catalysts produced with a 100 Hz and 20 Hz frequency, respectively. For further analysis of the HER activities, Figure S5b illustrates the recorded HER polarization curves for the Pd/C catalysts. The geometric activity (*GA*)

evaluated at -5 mV vs. RHE increases with decreasing applied frequency, starting with 0.72±0.10 mA cm_{geo}^{-2}, 0.75±0.04 mA cm_{geo}^{-2} and finally reaching 0.80±0.21 mA cm_{geo}^{-2} for the Pd/C catalysts prepared with 200 Hz, 100 Hz, and 20 Hz frequencies, respectively. Furthermore, we combine the tendencies of the *MA* and *SA* with the applied frequency during electrochemical erosion. Figure S5c depicts the dependence of the *SA* on the applied frequency. The *SA* of the Pd NPs synthesized with 200 Hz, 100 Hz, and 20 Hz frequency corresponds to 0.13±0.02 mA cm_{Pd}^{-2}, 0.14±0.01 mA cm_{Pd}^{-2}, and 0.14±0.04 mA cm_{Pd}^{-2}, respectively. Under consideration of the error bars, the *SA* is unaffected by frequency changes during synthesis. Figure S5d relates the *MA* to the applied frequency during electrochemical erosion. The *MAs* evaluated at -5 mV vs. RHE increases with decreasing applied frequency. For the Pd/C catalysts produced with 200 Hz, 100 Hz, and 20 Hz frequency, the *MA* corresponds to 48.2±7.0 mA mg_{Pd}^{-1}, 56.3±3.3 mA mg_{Pd}^{-1} and 66.3±17.5 mA mg_{Pd}^{-1}, respectively. It is well known that the concave and convex surface geometry resulting from the shape and defect states influence the catalytic activity for surface-sensitive reactions like the HER [iii,iv,v] or ORR.[vi,vii,viii,ix] Theoretical concepts correlate the adsorption energy of the reaction intermediate with the so-called coordination (*CN*) [x,xi,xii] or "generalized" coordination number ($\overline{CN}$) [xiii] of the catalyst atoms. Since Pd strongly binds the adsorbed hydrogen intermediate, it limits the HER activity through inhibited desorption of the gaseous H_2 molecules.[xiv] Therefore, HER activity is likely to be enhanced at atoms with slightly higher coordination numbers, which exist at cavity-like surface defects. This theoretical postulation matches the results of the Pd/C catalyst synthesized with 200 Hz, 100 Hz, and 20 Hz frequency. The defect density of the Pd NP significantly increases with a smaller frequency, as shown in the inset of Figure S2d, which displays a single Pd NP with a resolution scale bar of 5 nm. The blue dashed line indicates the "cloudy" shape of the NP, with arrows pointing toward concave-like defects. Those concavities of the 20 Hz Pd NP sample lead to the increased *MA* compared to the Pd NP with sharp edges formed by applying 200 Hz, as illustrated in Figure S5d.

(a) (b)

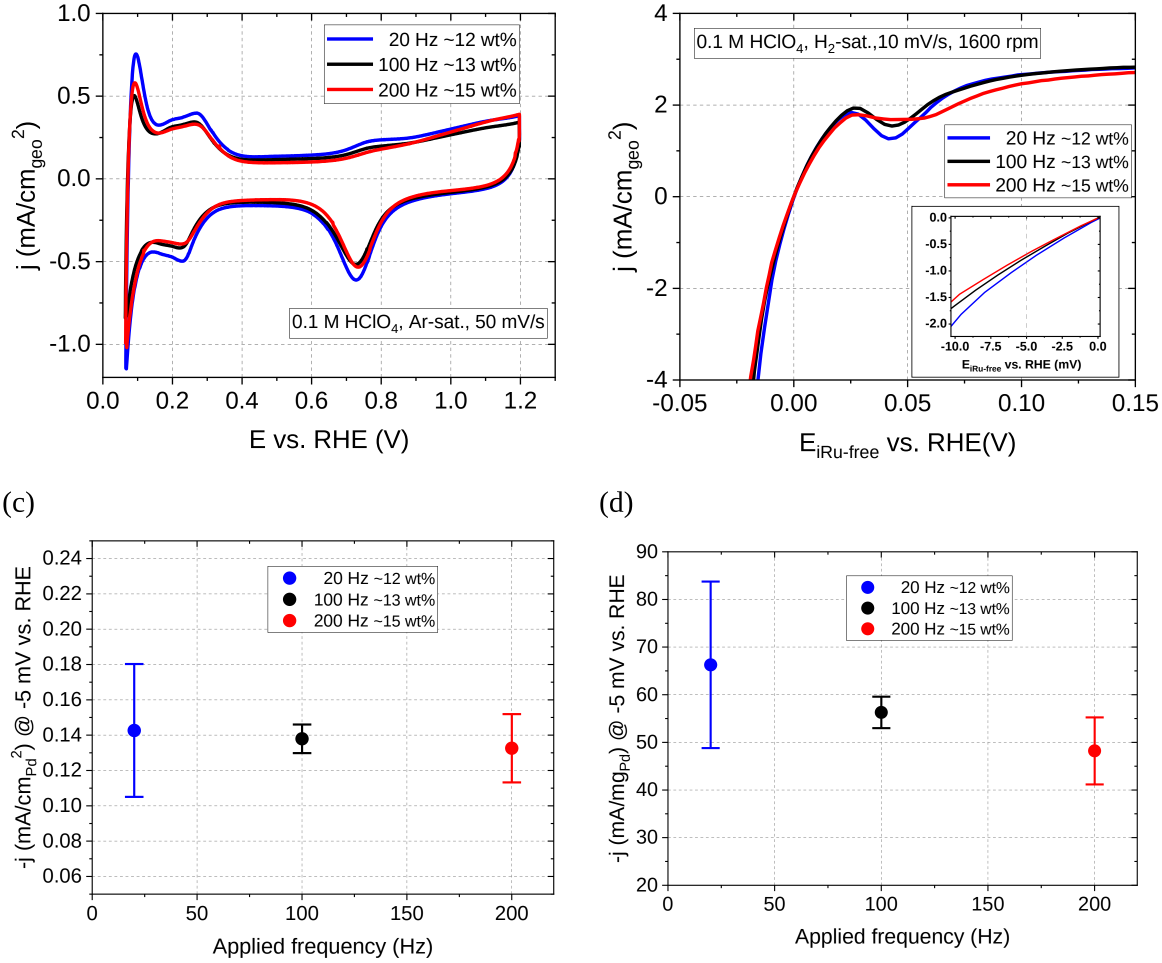

Figure S5. (a) CVs of Pd/C catalysts synthesized with 200 Hz, 100 Hz, and 20 Hz frequency. The curves were recorded in Ar-saturated 0.1 м $HClO_4$ at a scan rate of 50 mV s^{-1} and rotation speed of 400 rpm. (b) Characteristic iR_u-corrected HER polarization curves (cathodic scan) of Pd/C catalysts synthesized with 200 Hz, 100 Hz, and 20 Hz frequency. The curves were recorded in H_2-saturated 0.1 м $HClO_4$ at a scan rate of 10 mV s^{-1} and a rotation speed of 1600 rpm. Comparison of the (c) *SA* (d) *MA* evaluated at – 5 mV vs. RHE with the applied frequency during electrochemical erosion.

Table S1. Lattice parameter of the strained and non-strained Pd phases and their refined weight fractions in the total Pd/C catalyst batch.

**Pd/C batch**	**Lattice parameter** (phase 1; strained Pd) [nm]	**Lattice parameter** (phase 2; non-strained Pd) [nm]	**Weight ratio** (phase 1: phase 2) [wt%]
Pd/C 1 м $NaNO_3$	0.39285±0.00008	0.39008±0.00002	63:37
Pd/C 2 м $NaNO_3$	0.39224±0.00010	0.39044±0.00002	48:52
Pd/C 4 м $NaNO_3$	0.39309±0.00012	0.39082±0.00003	53:47
Pd/C 20 Hz	0.40025±0.00006	0.39012±0.00003	79:21
Pd/C 100 Hz	0.40107±0.00006	0.39005±0.00002	66:34
Pd/C 200 Hz	0.39950±0.00010	0.39021±0.00002	59:41

Table S2. Average crystallite size of the strained and non-strained Pd phases and the H_2O_2-XC72R Vulcan Carbon as obtained from XRD profiles.

**Pd/C batch**	**Average crystallite size** (phase 1; strained Pd) [nm]	**Average crystallite size** (phase 2; non-strained Pd) [nm]	**Average crystallite size** (H_2O_2-XC72R) [nm]
Pd/C 1 м $NaNO_3$	1.9	6.1	1.3
Pd/C 2 м $NaNO_3$	1.8	4.8	1.4
Pd/C 4 м $NaNO_3$	1.8	5.2	1.2
Pd/C 20 Hz	1.8	5.3	1.2
Pd/C 100 Hz	1.9	5.8	1.2
Pd/C 200 Hz	1.9	5.8	1.2

TGA analysis of Pd/C catalysts synthesized in 1 M, 2 M and 4 M $NaNO_3$

Figure S6 presents the TGA results obtained for the Pd/C catalysts synthesized in 1 M, 2 M and 4 M $NaNO_3$. A detailed description of the TGA measurement procedure can be found in the experimental section of the main manuscript.

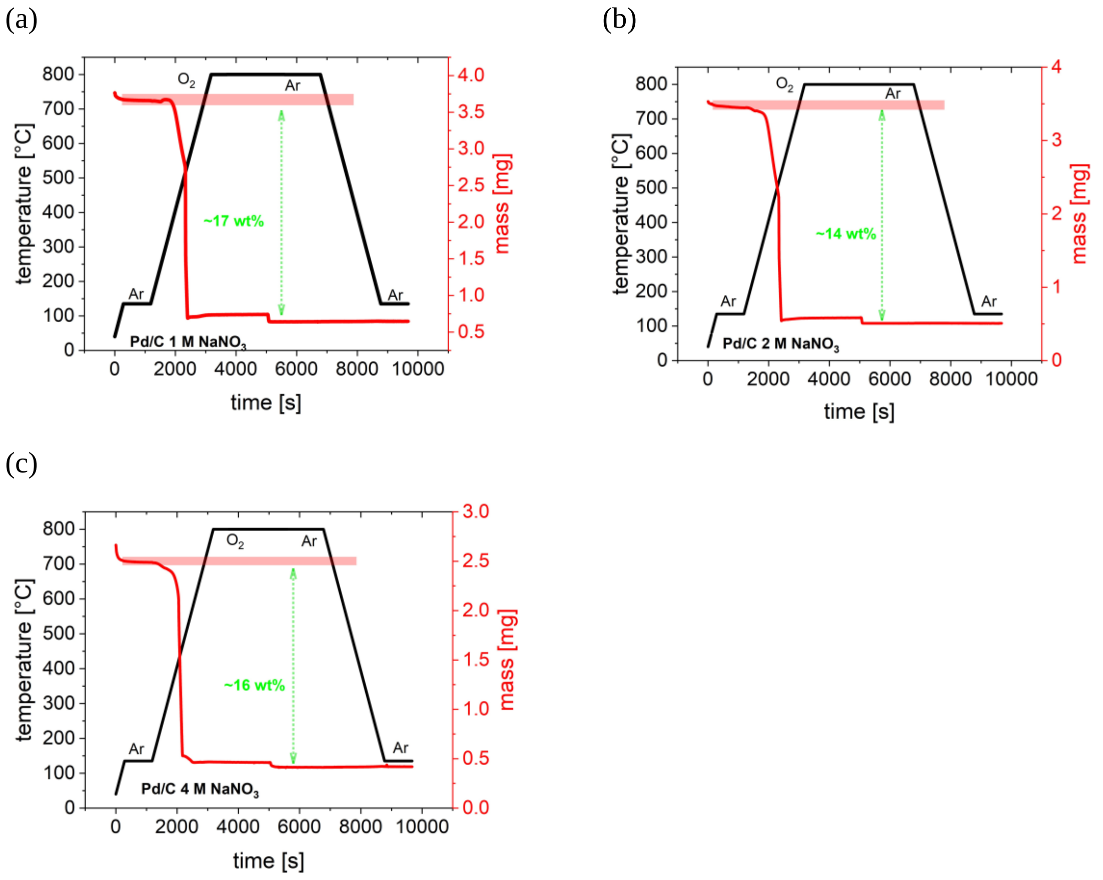

Figure S6. TGA measurements of the Pd/C catalysts synthesized in (a) 1 M, (b) 2 M, and (c) 4 M $NaNO_3$. The approximated weight loading was determined using the evaluation procedure from the STARe Software (V 16.30) from Mettler-Toledo.

---

[i] T. J. P. Hersbach, M. T. M. Koper, *Curr. Opin. Electrochem.* **2021**, *26*, 100653.

[ii] Z. Zhao, M. M. Flores Espinoza, J. Zhou, W. Xue, X. Duan, J. Miao, Y. Huang, *Nano Res.* **2019**, *12*, 1467, (Supplementary Material).

[iii] Y. Lian, C. Csoklich, D. McLaughlin, O. Schneider, A. S. Bandarenka, *ACS Appl. Mater. Interfaces* **2019**, *11*, 12476.

[iv] E. Mitterreiter, Y. Liang, M. Golibrzuch, D. McLaughlin, C. Csoklich, J. D. Bartl, A. Holleitner, U. Wurstbauer, A. S. Bandarenka, *npj 2D Mater. Appl.* **2019**, *3*.

[v] R. M. Kluge, R. W. Haid, I. E. L. Stephens, F. Calle-Vallejo, A. S. Bandarenka, *Phys. Chem. Chem. Phys.* **2021**, *23*, 10051.

[vi] F. Calle-Vallejo, M. D. Pohl, D. Reinisch, D. Loffreda, P. Sautet, A. S. Bandarenka, *Chem. Sci.* **2016**, *8*, 2283.

[vii] Y. Liang, D. McLaughlin, C. Csoklich, O. Schneider, A. S. Bandarenka, *Energy Environ. Sci.* **2019**, *12*, 351.

[viii] R. M. Kluge, E. Psaltis, R. W. Haid, S. Hou, T. O. Schmidt, O. Schneider, B. Garlyyev, F. Calle-Vallejo, A. S. Bandarenka, *ACS Appl. Mater. Interfaces* **2022**, *14*, 19604.

[ix] R. M. Kluge, R. W. Haid, A. Riss, Y. Bao, K. Seufert, T. O. Schmidt, S. A. Watzele, J. V. Barth, F. Allegretti, W. Auwärter, F. Calle-Vallejo, A. S. Bandarenka, *Energy Environ. Sci.* **2022**, *15*, 5181.

[x] F. Calle-Vallejo, M. T. M. Koper, *ACS Catal.* **2017**, *7*, 7346.

[xi] H. Li, Y. Li, M. T. M. Koper, F. Calle-Vallejo, *J. Am. Chem. Soc.* **2014**, *136*, 15694.

[xii] F. Calle-Vallejo, D. Loffreda, M. T. M. Koper, P. Sautet, *Nat. Chem.* **2015**, 7, 403.

[xiii] F. Calle-Vallejo, J. Tymoczko, V. Colic, Q. H. Vu, M. D. Pohl, K. Morgenstern, D. Loffreda, P. Sautet, W. Schuhmann, A. S. Bandarenka, *Science* **2015**, *350*, 185.

[xiv] J. K. Nørskov, T. Bligaard, A. Logadottir, J. R. Kitchin, J. G. Chen, S. Pandelov, U. Stimming, *J. Electrochem. Soc.* **2005**, *152*.
